# Relationship between social support status and mortality in a community-based population: a prospective observational study (Yamagata study)

**DOI:** 10.1186/s12889-020-09752-9

**Published:** 2020-10-29

**Authors:** Tsutomu Uzuki, Tsuneo Konta, Ritsuko Saito, Ri Sho, Tsukasa Osaki, Masayoshi Souri, Masafumi Watanabe, Kenichi Ishizawa, Hidetoshi Yamashita, Yoshiyuki Ueno, Takamasa Kayama

**Affiliations:** 1 Division of Nursing, Yamagata University Hospital, Yamagata, Japan; 2 Department of Public Health and Hygiene, Yamagata University Graduate School of Medical Science, 2-2-2, Iida-Nishi, Yamagata, 990-9585 Japan; 3 Global Center of Excellence Program Study Group, Yamagata University School of Medicine, Yamagata, Japan

**Keywords:** Cohort, Mortality, Social support

## Abstract

**Background:**

Social support, defined as the exchange of support in social relationships, plays a vital role in maintaining healthy behavior and mitigating the effects of stressors. This study investigated whether functional aspect of social support is related to 5-year mortality in health checkup participants.

**Methods:**

This study recruited 16,651 subjects (6797 males, 9854 females). Social support was evaluated using five-component questions: Do you have someone 1) whom you can consult when you are in trouble? 2) whom you can consult when your physical condition is not good? 3) who can help you with daily homework? 4) who can take you to hospital when you don’t feel well? and 5) who can take care of you when you are ill in bed? The association between the component of social support and all-cause and cardiovascular mortality was examined using Cox proportional hazard analysis.

**Results:**

The percentage of subjects without social support components was 7.7–15.0%. They were more likely to be male, non-elderly, and living alone. During the follow-up period, there were 166 all-cause and 38 cardiovascular deaths. Cox proportional analysis adjusted for confounders showed that only the lack of support for transportation to hospital was significantly associated with all-cause (hazard ratio [HR] 2.01, 95% confidence interval [CI] 1.26–3.05) and cardiovascular mortality (HR 3.30, 95% CI 1.41–6.87). These associations were stronger in males than females.

**Conclusion:**

This study showed that the lack of social support for transportation to the hospital was independently associated with all-cause and cardiovascular mortality in a community-based population.

## Background

In recent years, the social determinants of health (SDH) have attracted attention as an essential factor in the promotion of health and development of the disease. Social support/social networks, one of the components of SDH, is defined as “support that is exchanged in social relationships” and maintains healthy behaviors and mitigates the effects of stressors. However, the mechanisms by which social support is associated with health status are not sufficiently clarified and are still controversial [1]. One of the reasons for the difficulty of research on this subject may be the variety of measures to evaluate social support and outcomes [1, 2].

The Alameda study, a pioneering study on social support/social networks, showed that social engagement with people is associated with mortality, together with appropriate health habits [3]. Since then, many studies have reported that those who are socially isolated have higher mortality than those with many social support/networks in various populations including African American elderly women [4], local inhabitants in the United States [5–7], Finland [8], and Brazil [9]. These results indicate that the association between social support and mortality is commonly observed, irrespective of gender, age, and ethnicity. Furthermore, some reports revealed the association between social support and cardiovascular disease [8, 10]. In Japan, several studies have examined the relationship between various parameters of social support/networks and life prognosis in the general population [11–14]. However, it has not been examined the association between social support and the cause of death in detail.

Lakey and Cohen argue that social support research requires a theoretical perspective, and they raise the following categories of theoretical perspectives: (1) stress and coping, (2) social constructionist perspective, and (3) relationship perspective [15]. Cohen also speculates on the involvement of psychological mediators and neuroendocrine links to immune and cardiovascular function in how social support causes disease [16]. However, there are many properties in social support. Therefore, the study using different indicators may show different mechanisms or outcomes.

Berkman et al. used an integrated approach to understand how the structure and function of social relations and networks influence health outcomes [17]. However, it is difficult to distinguish social support and social networks clearly. In general, it seems that networks refer to the structural aspects of interpersonal relationships (such as the number of friends), while support refers to the functional aspects (such as help from friends) [2]. Most previous studies focused on social networks, which reflect structural aspects. On the other hand, previous Japanese studies on social support - the functional aspect - assessed its association with psychological status such as depression [18–20], but not mortality. We hypothesized that the functional aspects of social support play a role in the determination of life prognosis. Therefore, the present study examined the association between the functional aspects of social support and all-cause and cardiovascular mortality in the Japanese population, which have not often been examined before.

The Yamagata study aims to prospectively examine the association between genetic and environmental factors and common diseases and life prognosis in local inhabitants. In the present study, we used data from the Yamagata study to investigate whether social support is associated with all-cause and cardiovascular mortality in a community-based population.

## Methods

### Study subjects

The Yamagata study was conducted in seven cities (Yamagata, Kaminoyama, Sakata, Tendo, Higashine, Sagae, and Yonezawa) in Yamagata prefecture, Japan, with the support from the twenty-first Century Center of Excellence (COE) program and the Global COE program. Details of the Yamagata study have been described elsewhere [21]. This study’s target is the national health insurance-covered local inhabitants that are mainly agriculture, forestry and fisheries workers, self-employed, part-time workers, retirees, and unemployed. The number of potential subjects was 28,528 in this study. A total of 19,231 subjects aged 40 to 74 years provided written informed consent to participate in the baseline survey of the Yamagata Study from 2009 to 2015. Of the 19,231 who filled out the Yamagata study questionnaire, 2580 subjects who had missing answers in social support components and essential clinical information, including smoking, alcohol consumption, and medication, were excluded. The remaining 16,651 subjects (6797 males and 9854 females) were included in the final analysis of this study. The subjects have been followed from 2009 to the end of 2015.

### Baseline characteristics

A self-administered questionnaire was distributed to the study participants at the specific health checkup site and returned by postal mail. When distributing the questionnaire, we explained the outlines of the survey, including the voluntary nature of participation and the protection of personal information, and then the participants gave written informed consent. The Ethics Review Committee of the Faculty of the Medical Department of Yamagata University approved this study (approval No. 2018–464). This study was conducted based on the Declaration of Helsinki. The baseline questionnaire gathered information on social support using the following five questions because a previous study showed that these questions and answers were associated with depression in the Japanese population [19]: Do you have someone 1) whom you can consult when you are in trouble? 2) whom you can consult when your physical condition is not good? 3) who can help you in daily housework? 4) who can take you to a hospital when you don’t feel well? and 5) who can take care of you when you are ill in bed? The answer to each question was binary (yes/no).

In addition, basic information on smoking, alcohol consumption, cohabitation, and education history were collected. In the Japanese education system, compulsory education in elementary and junior high school is 9 years, up to high school, it is 10–12 years, and in college or beyond, it is 13 years or more. All citizens in Japan are required to have compulsory education, so it is unlikely that they have no formal education. Therefore, in this study, education history was divided into three groups of 9 years or less, 10 to 12 years, and 13 years or more. For lifestyle-related diseases, the presence of hypertension, diabetes, and dyslipidemia was ascertained based on the collected information on laboratory data and medications according to the definitions used in previous studies [21, 22].

### Classification of cause of death

Information on mortality was obtained from the death certificate. Cause of death was classified based on the International Statistical Classification of Diseases and Related Health Problems 10th Revision (ICD-10) code. Cardiovascular mortality was defined as the deaths due to the circulatory system (ICD-10 code I00-I99), such as acute myocardial infarction (I21), chronic ischemic heart disease (I25), cardiomyopathy (I42), heart failure (I50), subarachnoid hemorrhage (I60), intracerebral hemorrhage (I61) and cerebral infarction (I63). The cardiovascular disease was selected because previous studies had reported its association with social support [8, 10].

### Statistical analysis

Factors related to the components of social support were evaluated by logistic regression analysis. Survival analysis to examine the association between social support components and all-cause and cardiovascular mortality was performed using the Kaplan-Meier method. Independent associations of social support components with mortality were examined by Cox proportional hazards with adjustment for possible confounding factors including age, gender, education period, smoking, alcohol consumption, obesity, hypertension, diabetes, and dyslipidemia. A *p*-value of less than 0.05 was considered statistically significant. We used the statistical software JMP 14.2 for Windows (SAS Institute Japan Ltd., Tokyo, Japan) for all statistical analyses.

## Results

Baseline characteristics of the 16,651 study subjects (6797 males, 9854 females) are described in Table 1. The living conditions and prevalence of health-related factors and comorbidities were as follows: living alone (8.5%), smoking (12.5%), alcohol consumption (55.3%), hypertension (46.3%), diabetes (11.2%), dyslipidemia (57.3%), and obesity (24.0%).

**Table 1:** Baseline characteristics of study subjects

	Total subjects	Males	Females
Number	16,651	6797	9854
Age (years)	62.7 (8.4)	64.1 (7.9)	61.7 (8.5)
Body mass index (kg/m2)	23.0 (3.2)	23.6 (3.0)	22.6 (3.3)
Living alone (%)	8.5	8.0	8.9
Education period)			
≦ 9 years (%)	14.9	17.6	13.0
10–12 years (%)	55.2	55.5	55.2
≧ 13 years (%)	29.9	26.9	32.0
Smoking (%)	12.5	22.8	5.1
Alcohol consumption (%)	55.3	79.9	38.0
Hypertension (%)	46.3	55.9	39.7
Diabetes (%)	11.2	20.7	9.2
Dyslipidemia (%)	57.3	55.7	58.7
Obesity (%)	24.0	29.1	20.5

Mean (SD)

### Status of social support

Table 2 shows the results of the five component social support questions. Overall, the prevalence of the lack of someone 1) whom you can consult when you are in trouble was 12.7%; 2) whom you can consult when your physical condition is not good was 10.6%; 3) who can help you in daily homework was 15.0%; 4) who can take you to a hospital when you don’t feel well was 7.7%; 5) who can take care of you when you are ill in bed was 11.4%. For most of the five components, men had a higher prevalence of lacking social support than women. Logistic regression analysis of factors related to a lack of social support components showed that male sex, age under 65, and living alone were independently associated with the lack of almost all five social support components (Table 3). Further, smoking was associated with the lack of someone 3) who can help you in daily homework and 5) who can take care of you when you are ill in bed. In contrast, alcohol consumption was associated with having someone 1) whom you can consult when you are in trouble, 2) whom you can consult when your physical condition is not good, and 5) who can take care of you when you are ill in bed. Comorbidity factors were not associated with the lack of social support components.

**Table 2:** The prevalence of the lack of social support components

	Total subjects	Males	Females
1) No one whom you can consult when you are in trouble (%)	12.7	19.1	8.2
2) No one whom you can consult when your physical condition is not good (%)	10.6	15.1	7.6
3) No one who can help you in daily housework (%)	15.0	19.1	12.3
4) No one who can take you to a hospital when you don’t feel well (%)	7.7	9.5	6.4
5) No one who can take care of you when you are ill in bed (%)	11.4	10.7	12.1

**Table 3:** Multivariate logistic regression analysis to predict the lack of social support components

	1) Consult at trouble	2) Consult at physical condition	3) Support for daily housework	4) Support for transportation to hospital	5) Take care of you when sick
	OR (95%CI)	OR (95%CI)	OR (95%CI)	OR (95%CI)	OR (95%CI)
Male gender	2.78 (2.45–3.15)	2.53 (2.21–2.90)	1.84 (1.64–2.07)	1.89 (1.61–2.22)	0.92 (0.80–1.05)
< 65 years	1.23 (1.10–1.39)	1.37 (1.22–1.54)	1.12 (1.01–1.25)	1.54 (1.34–1.78)	1.35 (1.19–1.54)
Living alone	1.87 (1.60–2.18)	2.48 (2.12–2.90)	4.71 (4.13–5.34)	10.7 (9.23–12.4)	10.2 (8.90–11.6)
Education period					
≦ 9 years (%)	Reference	Reference	Reference	Reference	Reference
10–12 years (%)	1.06 (0.91–1.24)	1.09 (0.91–1.30)	0.89 (0.77–1.03)	0.93 (0.76–1.15)	0.96 (0.80–1.15)
≧ 13 years (%)	1.14 (0.96–1.35)	1.21 (1.01–1.47)	0.95 (0.81–1.12)	1.08 (0.86–1.35)	1.13 (0.93–1.38)
Smoking	1.10 (0.95–1.27)	1.08 (0.92–1.26)	1.18 (1.03–1.36)	1.17 (0.93–1.36)	1.34 (1.13–1.60)
Alcohol consumption	0.81 (0.72–0.91)	0.84 (0.74–0.95)	0.90 (0.81–1.01)	0.88 (0.75–1.03)	0.84 (0.74–0.96)
Hypertension	1.04 (0.93–1.16)	1.15 (1.02–1.30)	0.98 (0.88–1.09)	1.01 (0.88–1.17)	1.00 (0.88–1.13)
Diabetes	1.06 (0.91–1.24)	1.02 (0.85–1.21)	1.11 (0.91–1.12)	1.01 (0.81–1.24)	0.99 (0.82–1.19)
Dyslipidemia	1.02 (0.91–1.13)	1.00 (0.89–1.12)	0.99 (0.89–1.10)	0.90 (0.78–1.04)	0.88 (0.78–0.99)
Obesity	1.06 (0.94–1.19)	1.08 (0.94–1.23)	1.02 (0.90–1.14)	0.96 (0.81–1.13)	1.08 (0.94–1.24)

*CI* confidence interval, *OR* odds ratio

### Association between social support and mortality

**Fig. 1:**
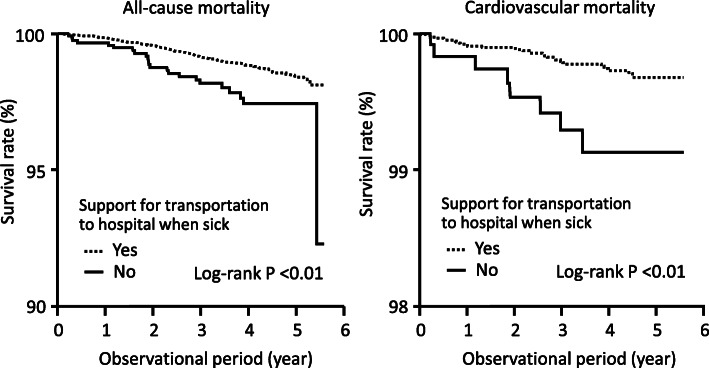
Association between support for transportation to hospital and mortality

During the 5-year follow-up period (median 3.4 years), there were 166 total deaths (116 males, 50 females) and 38 cardiovascular deaths (27 males, 11 females). First, we performed a Kaplan-Meier survival analysis of the association between each component of social support and mortality. A significant association with all-cause and cardiovascular mortality was observed only with the transport support to hospital, but not with the other components. The Kaplan-Meier analysis in Fig. 1 compared the survival curves between the two groups (yes/no) regarding the presence of “Support for transportation to hospital when sick”, based on the answer to question 4). It showed that the all-cause and cardiovascular mortality of those without transport support to the hospital were significantly higher than those with such support.

Similarly, unadjusted Cox proportional hazard analysis showed that only a lack of support for transportation to hospital had a significant association with all-cause mortality and cardiovascular mortality (HR, 2.01, 95% CI, 1.26–3.05 for all-cause mortality, and HR, 3.30, 95% CI, 1.41–6.87 for cardiovascular mortality) (Table 4). These associations remained significant after adjustment for possible confounding factors (HR, 1.88, 95% CI, 1.15–2.93 for all-cause mortality, and HR, 2.73, 95% CI, 1.09–5.95 for cardiovascular mortality) (Table 4). In this multivariate model, the independent risk factors for all-cause mortality, other than support for transport to the hospital, were older age, men, diabetes, and smoking. On analysis by sex, the significant association with all-cause mortality was observed in males (adjusted HR, 2.07, 95% CI, 1.18–3.40) but not in females (adjusted HR, 1.46, 95%CI, 0.44–3.62). We further examined the association between all-cause mortality and structural aspects of social support (living alone) using the same multivariate model. However, this factor did not show an independent association with all-cause mortality.

**Table 4:** The association between the lack of social support components and mortality

	Unadjusted		Adjusted^a^	
	HR (95%CI)	*P* value	HR (95%CI)	*P* value
All-cause mortality				
1) No one whom you can consult when you are in trouble	1.44 (0.94–2.12)	0.09	1.15 (0.73–1.74)	0.53
2) No one whom you can consult when your physical condition is not good	1.27 (0.77–1.94)	0.34	1.18 (0.71–1.85)	0.50
3) No one who can help you in daily housework	1.38 (0.93–2.01)	0.11	1.18 (0.78–1.76)	0.41
4) No one who can take you to a hospital when you don’t feel well	2.01 (1.26–3.05)	< 0.01	1.88 (1.15–2.93)	0.01
5) No one who can take care of you when you are ill in bed	1.02 (0.60–1.61)	0.94	1.09 (0.62–1.77)	0.76
Cardiovascular mortality				
1) No one whom you can consult when you are in trouble	1.88 (0.80–3.90)	0.14	1.38 (0.55–3.02)	0.47
2) No one whom you can consult when your physical condition is not good	1.31 (0.45–3.05)	0.59	0.94 (0.28–2.39)	0.90
3) No one who can help you in daily housework	1.54 (0.66–3.20)	0.30	1.18 (0.47–2.57)	0.70
4) No one who can take you to a hospital when you don’t feel well	3.30 (1.41–6.87)	< 0.01	2.73 (1.09–5.95)	0.03
5) No one who can take care of you when you are ill in bed	1.27 (0.43–2.94)	0.64	1.11 (0.33–2.83)	0.84

*CI* confidence interval, *HR* hazard ratio

^a^Adjusted for age, gender, education period, hypertension, diabetes, dyslipidemia, obesity, alcohol consumption, smoking

## Discussion

This study in a Japanese community-based population showed that the prevalence of a lack of social support was higher in men, non-elderly, and those living alone. Furthermore, a lack of support for transportation to hospital when sick was independently associated with all-cause and cardiovascular mortality, especially in males.

Previous studies using the same questionnaire reported that the proportion of people without social support is low (1.3–8.2%) in the suburbs [23] and high (22.1–39.1%) in urban areas [18]. Our present participants live in middle-sized cities, and the proportion of participants with a lack of social support (7.7–15.0%) is in the middle of ranges in previous studies. These observations indicate that the availability of social support is related to the characteristics of the region. This study also showed that people living alone were less likely to have social support. This observation would appear consistent with previous findings that isolation leads to a lack of information and resources, a lack of support, and a decrease in self-efficacy and self-esteem, and as a result, that illness tends to occur [3, 24]. Our study also revealed that non-elderly is less likely to have social support. It has been reported that social support of the local community is decreasing with increasing age in Japan [25]. Compared to older people who are close to each other, it might be more difficult for younger people who live a work-centered life to obtain social support. These results indicate that the status of social support is affected by individual factors such as gender and age and by environmental factors such as cohabitation and neighborhood relations in the entire community.

In this study, longitudinal analysis disclosed that a lack of support for transportation to the hospital when sick was significantly associated with all-cause and cardiovascular mortality, even after adjustment for background factors. Furthermore, this association was stronger in males than in females. This finding suggests that a lack of support for transportation to hospital may be a risk for mortality, independently of comorbidities and age, especially in males.

One possible reason for this association is that transportation support may lead to rapid treatment of critical cardiovascular diseases, such as stroke and myocardial infarction. In cardiovascular disease, the speed of treatment has a significant effect on prognosis. One study reported that in the patients with stroke or transient ischemic attack, cohabitation was associated with improved time to arrival at hospital in men only [26]. Another study reported that living alone confers a risk of all-cause and cardiovascular death in men, but not in women [10]. These findings suggest that support for transportation to hospital when sick, one of the social supports, might affect mortality by shortening time to receiving treatment for critical conditions.

To assess social support, three major perspectives (stress and coping, social constructionist, and relationship) were proposed by Lakey and Cohen et al. [15]. In the current study, we did not directly assess the subjects’ level of stress, self-esteem, companionship and intimacy with supporters. However, this study’s social support questionnaire mainly reflects the companionship and intimacy between the study subjects and their supporters in their daily lives, so this study is largely the relationship perspective-based. Our finding suggests the importance of the relationship between individuals on health outcomes.

The strength of this study is its large sample size and adjustment for various confounding factors, which appear to warrant the robustness of the results. Several limitations of the study also warrant mention. First, the functional aspect of social support was evaluated from simple questions and binary answers. We did not take into account the degree of each support. Second, we performed multivariate analyses adjusting for various established risk factors. However, other confounding factors may still have been present. Further, because of the small number of events, we did not include analyses with the additional adjustment, such as structural factors of social support (living alone). Third, the study subjects were participants in a community-based annual health checkup and questionnaire survey. Compared to the general population, they might have been more health-conscious and had a higher level of social activity. Therefore, a selection bias might be present. Fourth, the study was conducted under an observational design and cannot determine causation in the relationship between social support and mortality. Fifth, social support status was evaluated only once at baseline and might have changed during the follow-up period.

## Conclusions

This study evaluated the functional aspects of social support, which to date, has received little research interest. Our results identified an association between support for transportation to the hospital - a functional aspect of social support - and mortality in a community-based population. In modern societies, social support is admittedly challenging to maintain; nevertheless, the establishment and utilization of social support is crucial to improving life prognosis in local communities. The finding of the current study that social support is associated with medical outcomes also suggests a possibility that such support contributes to the maintenance of social capital via preventing the deterioration of living conditions and could be one of the broad forms of support that lower delinquency and crime in the community in the long run.

## Data Availability

The datasets generated during and/or analyzed during the current study are not publicly available due to ethical reasons, but are available from the corresponding author on reasonable request.

## Abbreviations

BMI: Body mass index
CI: Confidence interval
HR: Hazard ratio
ICD-10: International Statistical Classification of Diseases and Related Health Problems 10th Revision
SDH: Social determinants of health
